# Acute microcirculatory effects of medium frequency versus high frequency neuromuscular electrical stimulation in critically ill patients - a pilot study

**DOI:** 10.1186/2110-5820-3-39

**Published:** 2013-12-19

**Authors:** Epameinondas Angelopoulos, Eleftherios Karatzanos, Stavros Dimopoulos, Georgios Mitsiou, Christos Stefanou, Irini Patsaki, Anastasia Kotanidou, Christina Routsi, George Petrikkos, Serafeim Nanas

**Affiliations:** 1 First Critical Care Department, National and Kapodistrian University of Athens School of Medicine, Evangelismos General Hospital, Ypsilantou 45-47, Athens 106 75, Greece; 2 Fourth Department of Internal Medicine, National and Kapodistrian University of Athens School of Medicine, Attikon University General Hospital, Rimini 1, Athens 124 62, Greece

**Keywords:** Microcirculation, Neuromuscular electrical stimulation, Near-infrared Spectroscopy, ICU-acquired weakness prevention, Tissue oxygen saturation

## Abstract

**Background:**

Intensive care unit-acquired weakness (ICUAW) is a common complication, associated with significant morbidity. Neuromuscular electrical stimulation (NMES) has shown promise for prevention. NMES acutely affects skeletal muscle microcirculation; such effects could mediate the favorable outcomes. However, optimal current characteristics have not been defined. This study aimed to compare the effects on muscle microcirculation of a single NMES session using medium and high frequency currents.

**Methods:**

ICU patients with systemic inflammatory response syndrome (SIRS) or sepsis of three to five days duration and patients with ICUAW were studied. A single 30-minute NMES session was applied to the lower limbs bilaterally using current of increasing intensity. Patients were randomly assigned to either the HF (75 Hz, pulse 400 μs, cycle 5 seconds on - 21 seconds off) or the MF (45 Hz, pulse 400 μs, cycle 5 seconds on - 12 seconds off) protocol. Peripheral microcirculation was monitored at the thenar eminence using near-infrared spectroscopy (NIRS) to obtain tissue O_2_ saturation (StO_2_); a vascular occlusion test was applied before and after the session. Local microcirculation of the vastus lateralis was also monitored using NIRS.

**Results:**

Thirty-one patients were randomized. In the HF protocol (17 patients), peripheral microcirculatory parameters were: thenar O_2_ consumption rate (%/minute) from 8.6 ± 2.2 to 9.9 ± 5.1 (*P* = 0.08), endothelial reactivity (%/second) from 2.7 ± 1.4 to 3.2 ± 1.9 (*P* = 0.04), vascular reserve (seconds) from 160 ± 55 to 145 ± 49 (*P* = 0.03). In the MF protocol: thenar O_2_ consumption rate (%/minute) from 8.8 ± 3.8 to 9.9 ± 3.6 (*P* = 0.07), endothelial reactivity (%/second) from 2.5 ± 1.4 to 3.1 ± 1.7 (*P* = 0.03), vascular reserve (seconds) from 163 ± 37 to 144 ± 33 (*P* = 0.001). Both protocols showed a similar effect. In the vastus lateralis, average muscle O_2_ consumption rate was 61 ± 9%/minute during the HF protocol versus 69 ± 23%/minute during the MF protocol (*P* = 0.5). The minimum amplitude in StO_2_ was 5 ± 4 units with the HF protocol versus 7 ± 4 units with the MF protocol (*P* = 0.3). Post-exercise, StO_2_ increased by 6 ± 7 units with the HF protocol versus 5 ± 4 units with the MF protocol (*P* = 0.6). These changes correlated well with contraction strength.

**Conclusions:**

A single NMES session affected local and systemic skeletal muscle microcirculation. Medium and high frequency currents were equally effective.

## Background

The development of muscle weakness is a frequent occurrence in ICU patients. Both nerve and muscle dysfunction can be responsible for this clinical condition, variably known as critical illness neuropathy, critical illness myopathy, and ICU-acquired weakness (ICUAW) among other terms, and is presumably a complication of sepsis [1,2]. Patients with ICUAW are prone to have difficult weaning from mechanical ventilation and to have a longer duration of ICU stay, with all the associated risks [3,4]. Even after discharge, patients can have severely impaired muscle force for months or years, with significant repercussions on their quality of life [5,6].

With no treatment available, emphasis has been placed on finding effective preventive measures. Early patient mobilization has been increasingly adopted, since it has been shown to produce better outcomes with regard to muscle strength and functionality [7]. Since active mobilization is not feasible for many ICU patients, due to either their illness or sedation, neuromuscular electrical stimulation (NMES) has been proposed as an alternative method of exercise and mobilization. This approach was based on clinical experience in patients with chronic obstructive pulmonary disease (COPD) [8,9] and chronic heart failure [10]. Initial application of NMES in septic patients using electrical currents with frequencies in the range of medium (45 to 50 Hz) to high (100 Hz) has shown encouraging results in preventing loss of muscle mass and preserving strength in the critically ill [11-15].

In addition, a single session of NMES-induced exercise using medium frequency current (45 Hz) has been shown to acutely induce systemic changes in skeletal muscle microcirculatory function, as assessed with near-infrared spectroscopy (NIRS). It has been suggested that these changes could mediate the beneficial effects of NMES on the skeletal musculature [16]. It is not known if high frequency NMES has comparable effects on the systemic microcirculation of skeletal muscle. As a result, the NMES characteristics of choice for critically ill patients are still undefined, and a comparison of the effects on skeletal muscle microcirculation is an important component in the assessment of efficacy of the different types.

The primary aim of this study was to compare the changes in systemic and local skeletal muscle microcirculation during a single session of medium frequency NMES and a single session of high frequency NMES on the lower limbs of critically ill patients. As secondary objectives, the effects of NMES on the microcirculation specifically in the presence of ICUAW were examined.

## Methods

### Patient selection

Two distinct groups of patients were enrolled in the study. Patients eligible for the first group (referred from this point on as the ‘acute group’) had to meet the diagnostic criteria for the systemic inflammatory response syndrome (SIRS) or sepsis of any severity [17] for a minimum of three and a maximum of five days at the day of the session. These time constraints were selected because they represent the minimum time period in which ICUAW can develop [18]. Exclusion criteria were age under 18 years, pregnancy, pre-existing neuromuscular disease, conditions that limit lower limb mobility (for example, burns, fractures), pacemakers, and presence of edema or subcutaneous fat that interfered with the application of NMES or with the assessment of the microcirculation.

The second group consisted of patients who had developed clinically manifest ICUAW. These will subsequently be referred to as the ‘ICUAW group’. The diagnosis of ICUAW was made on clinical grounds after the patients had been awaken from sedation and their orientation and cooperation had been ascertained, following a protocol described previously [11,19].

### Study design

This was a prospective, randomized pilot study. The study protocol was approved by the Scientific Committee of Evangelismos Hospital. Written informed consent to participate in the study was provided by all patients or their next of kin.

In all patients, demographic and clinical data were recorded, as were medications, sedative agents and ventilation mode used on the day of the NMES session. Vital signs, arterial oxygenation, central venous oxygen saturation and lactate levels were obtained before and after the NMES session. Creatine kinase (CK) levels were monitored for four days following the application of NMES.

### NMES application and evaluation of muscle contractions

NMES was applied to both lower limbs of the patients. An electronic stimulator (Cefar Rehab Pro, Cefar Medical AB, Malmö, Sweden) was used for the application. After appropriate skin preparation, rectangular adhesive electrodes (9 × 5 cm) were placed over the motor points of the vastus lateralis, vastus medialis and peroneus longus muscles.

Patients were randomly assigned to one of two NMES protocols. The HF (high frequency) protocol consisted of symmetric, biphasic, trapezoid pulses at 75 Hz, with pulse duration of 400 μs, duty cycle of 5 seconds on and 21 seconds off, ramp-up time of 1.5 seconds and ramp-down time of 0.8 seconds. The MF (medium frequency) protocol consisted of symmetric, biphasic, trapezoid pulses at 45 Hz, with pulse duration of 400 μs, duty cycle of 5 seconds on and 12 seconds off, ramp-up time of 1.5 seconds and ramp-down time of 0.8 seconds. The duration of each session was 30 minutes for both protocols, with an additional 5-minute warm-up and a 5-minute recovery phase using 10 Hz current of 400 μs pulse duration.

The specific waveforms were selected because they have been shown to maximize force and delay fatigue, compared with other forms such as the sinusoidal [20,21]. Frequencies and duty cycles were combined based on results from a study which specifically investigated optimal combinations of NMES settings [22].

Current intensity was continually adjusted during each session. It has been known that when a muscle group is stimulated with constant intensity, the generated torque gradually decreases [22,23]. In a set of preliminary trials, we verified that the need to increase the administered intensities is also true for the two NMES protocols under investigation (data not shown). As a result, in alert patients, current intensity was initially set to the maximal tolerated level, and was increased by 10% (or less, if the patient felt discomfort) every three minutes. In sedated patients, after the warm-up, the intensity that produced a maximal response was determined, and the 60% of that value was used as the starting point. In this case also current intensity was increased by 10% every three minutes.

Muscle contractions of the quadriceps muscles were graded by one of the researchers, who had no knowledge of which protocol was being used. A score was given according to the following scale: 0 = no contraction, 1 = palpable contraction, 2 = visible contraction, 3 = slight knee extension against gravity, 4 = full knee extension [24].

### Near-infrared spectroscopy and the vascular occlusion technique

NIRS is used to estimate the concentration of specified chromophores in a given tissue volume. In clinical settings, the parameter of interest is the degree of hemoglobin oxygenation, termed tissue oxygen saturation (StO_2_). This quantity is evaluated at the microcirculatory compartment, according to principles described previously [25,26].

We used a continuous-wave spectrometer (Hutchinson InSpectra Model 650, Hutchinson Technology, Hutchinson, MN, USA). This device emits light in four wavelengths and uses a wide gap second-derivative method to estimate StO_2_ with a measurement depth of 15 mm [27].

NIRS measurements were continuously recorded at two distinct sites. A probe was placed over the thenar eminence in order to capture the effects of NMES on the systemic microcirculation. In addition, the vascular occlusion technique (VOT) was applied in order to reveal features of the microcirculation that the simple monitoring cannot provide. The VOT consists of applying a standardized ischemic stimulus to the upper extremity by inflating a pneumatic cuff to 50 mmHg above the systolic pressure and maintaining it for three minutes. After that the cuff is released and perfusion is reestablished.

During the ischemic phase, the StO_2_ signal decreases. Its slope, expressed in % units per minute (downslope, %/minute), has been shown to be a proxy for local muscle O_2_ consumption rate [28]. During the reperfusion phase, the StO_2_ signal rapidly increases, usually to a value higher than the initial one, and then gradually returns to baseline. The initial increase of the signal, expressed in % units per second (upslope, %/second), is an index of endothelial reactivity, while the duration of the hyperemic phase reflects vascular reserve [29].

A second NIRS probe was placed over the vastus lateralis muscle, in a randomized manner with regards to side. The parameters that were evaluated were the StO_2_ desaturation slope (ΔStO_2slope_), which was calculated as the average of the slopes of the StO_2_ signal in each contraction and which serves as an index of muscle O_2_ consumption [30,31], the StO_2_ minimum amplitude (ΔStO_2min_), which is the difference between the minimum StO_2_ value during a contraction and baseline StO_2_ and is an index of O_2_ demand relative to O_2_ supply [31,32], and the StO_2_ maximum amplitude (ΔStO_2max_), which is the difference between the maximum StO_2_ value during the recovery period and baseline StO_2_, with higher values indicating increased O_2_ supply relative to O_2_ demand [32].

All NIRS measurements were stored and analyzed off-line using the proprietary software Hutchinson InSpectra Analysis v. 2.4 (Hutchinson Technology, Hutchinson, MN, USA), running in Matlab v. 7.0 (The MathWorks Inc., Natick, MA, USA).

### Statistical analysis

Continuous variables are expressed as mean ± standard deviation. Ordinal variables are expressed as median and interquartile range (IQR). Comparisons were made using Mann–Whitney *U*-test for independent samples and Wilcoxon’s signed rank test for dependent samples. Correlations were estimated using Spearman’s rank correlation coefficient. Categorical variables were compared with Fisher’s exact test. *P* values < 0.05 were considered statistically significant.

Linear regression models were used to evaluate the impact of predictor variables on continuous outcome variables. Model comparison was made using Akaike’s Information Criterion (AIC) to evaluate which predictors were more closely associated with the outcome [33,34].

All analyses were performed using IBM SPSS 20 for Windows (IBM Corp., Armonk, NY, USA).

## Results

A total of 31 patients were included in the study (26 men, age 59 ± 11 years, with Acute Physiology and Chronic Health Evaluation II (APACHE II) score 18 ± 6 and Sequential Organ Failure Assessment (SOFA) score 7 ± 2 on the day of the NMES session). Basic characteristics of patients for the two NMES protocols are shown in Table 1.

**Table 1:** Basic characteristics of the 31 critically ill patients by neuromuscular electrical stimulation (NMES) protocol

	**HF protocol**	**MF Protocol**
	**(n = 17)**	**(n = 14)**
Age (years)	57 ± 12	63 ± 12
Sex	15♂/2♀	11♂/3♀
SOFA score at admission	7 ± 3	7 ± 4
APACHE II score at admission	21 ± 7	18 ± 8
SOFA score at NMES session	7 ± 2	7 ± 3
APACHE II score at NMES session	16 ± 7	19 ± 6
Mechanical ventilation		
VC/PS/VM	5/7/5	6/5/3
Sedation (n)	9	11
Admission diagnosis		
Respiratory infection (n)	4	3
Sepsis (n)	3	3
COPD exacerbation (n)	0	1
Head trauma (n)	3	3
Multiple trauma (n)	3	1
Drug overdose (n)	1	0
Intracerebral hemorrhage (n)	2	2
Pancreatitis (n)	1	0
Patients with ICUAW (n)	7	5

APACHE II = Acute Physiology and Chronic Health Evaluation II; HF = high frequency; ICUAW = intensive care unit-acquired weakness; MF = medium frequency; PS = pressure support; SOFA = Sequential Organ Failure Assessment; VC = volume control; VM = Venturi mask.

Hemodynamic parameters were mildly affected during NMES application: heart rate, systolic and diastolic blood pressure were slightly increased. This effect was observed with both protocols and had no clinical importance. Blood lactate levels showed a moderate increase in the two NMES protocols (Table 2). In every occasion, the magnitude of these effects was similar in the two protocols.

**Table 2:** Clinical and laboratory data of patients before and after the neuromuscular electrical stimulation (NMES) session

	**HF protocol (n = 17)**			**MF protocol (n = 14)**		
	**Before NMES**	**After NMES**	***P***	**Before NMES**	**After NMES**	***P***
Hemodynamic parameters						
Systolic BP (mmHg)	125 ± 19	130 ± 18	0.17	128 ± 19	135 ± 25	0.06
Diastolic BP (mmHg)	71 ± 11	74 ± 12	0.19	62 ± 9	65 ± 11	0.25
Heart rate (min^-1^)	97 ± 14	100 ± 17	0.18	93 ± 19	100 ± 18	0.01
Respiratory parameters						
Respiratory rate (min^-1^)	22 ± 7	23 ± 6	0.4	22 ± 6	25 ± 9	0.04
SpO_2_ (%)	98 ± 1	98 ± 2	0.5	98 ± 1	98 ± 2	0.3
PaO_2_ (mmHg)	125 ± 39	121 ± 40	0.1	128 ± 30	121 ± 28	0.2
PaCO_2_ (mmHg)	36 ± 4	36 ± 9	0.9	36 ± 9	32 ± 3	0.6
ScvO_2_ (%)	74 ± 8	77 ± 6	0.08	78 ± 7	79 ± 5	0.6
Other						
Temperature (°C)	37.1 ± 1.5	37.2 ± 1.5	0.5	37.7 ± 1,1	37.5 ± 1,1	0.04
Lactate (mEq/mL)	1.3 ± 0.4	1.5 ± 0.5	0.06	1.0 ± 0.5	1.4 ± 0.6	0.007

HF, high frequency; MF, medium frequency; PaCO_2_, partial pressure of carbon dioxide in arterial blood; PaO_2_, partial pressure of oxygen in arterial blood; SpO_2_, oxygen saturation; ScvO_2_, central venous oxygen saturation.

NMES was well tolerated by the patients, and no adverse effects were noted during the application of NMES or in the follow-up period. CK levels did not increase during the four days following the NMES session (data not shown).

### Systemic microcirculation

The parameters of the systemic microcirculation before and after the NMES session for the HF and the MF protocol are shown in Table 3. StO_2_ decreased very slightly, by 1.6 ± 2.9 units and 0.9 ± 3.7 units, respectively. StO_2_ downslope increased in both protocols, by 1.2 ± 3.7%/minute and 1.1 ± 1.7%/minute, respectively, although these changes did not reach statistical significance. StO_2_ upslope also increased by 0.56 ± 0.83%/second and 0.52 ± 0.92%/second. This effect did not differ between the two protocols (*P* = 0.9). Duration of hyperemia was also affected: the time to baseline was reduced by 15 ± 26 seconds and 19 ± 16 seconds. Again this reduction did not differ significantly between the two protocols (*P* = 0.6).

**Table 3:** Changes in microcirculatory parameters monitored by near-infrared spectroscopy and use of a vascular occlusion test in the two neuromuscular electrical stimulation (NMES) protocols

	**HF protocol (n = 17)**			**MF protocol (n = 14)**		
**Microcirculatory parameter**	**Before**	**After**	***P***	**Before**	**After**	***P***
Tissue oxygenation (StO_2_)	79 ± 10	77 ± 11	0.06	76 ± 10	75 ± 11	0.6
StO_2_ downslope (%/minute)	8.6 ± 2.2	9.9 ± 5.1	0.08	8.8 ± 3.8	9.9 ± 3.6	0.07
StO_2_ upslope (%/second)	2.7 ± 1.4	3.2 ± 1.7	0.04	2.5 ± 1.4	3.1 ± 1.7	0.03
Time to baseline (seconds)	160 ± 55	145 ± 49	0.03	163 ± 37	144 ± 33	0.001

Legend: HF, high frequency; MF, medium frequency: StO_2_, tissue oxygen saturation.

**Figure 1:**
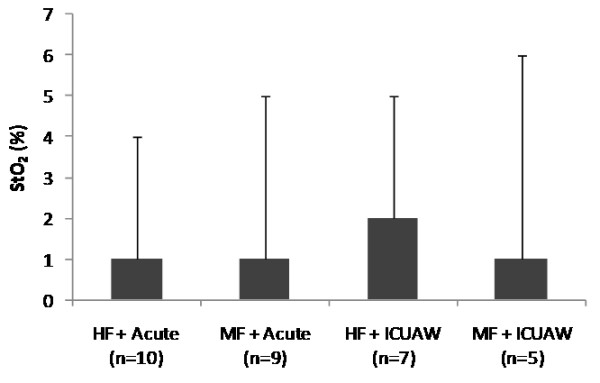
**Change scores in thenar muscle tissue oxygen saturation (StO**_**2**_**) as measured by near-infrared spectroscopy (NIRS) for each neuromuscular electrical stimulation (NMES) protocol and patient group combination.** Scores are calculated as before minus after values.

**Figure 2:**
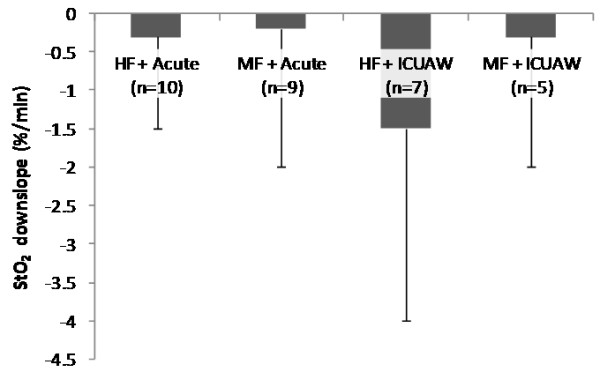
**Change scores in thenar muscle oxygen consumption rate (tissue oxygen saturation (StO**_**2**_**) downslope) as measured by near-infrared spectroscopy (NIRS) during a vascular occlusion test for each neuromuscular electrical stimulation (NMES) protocol and patient group combination.** Scores are calculated as before minus after values.

**Figure 3:**
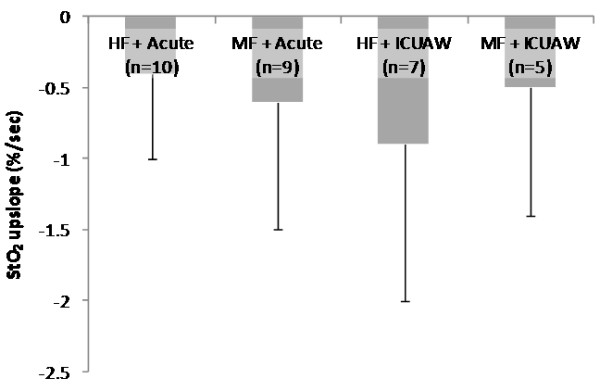
**Change scores in thenar muscle endothelial reactivity (tissue oxygen saturation (StO**_**2**_**) upslope) as measured by near-infrared spectroscopy (NIRS) during a vascular occlusion test for each neuromuscular electrical stimulation (NMES) protocol and patient group combination.** Scores are calculated as before minus after values.

**Figure 4:**
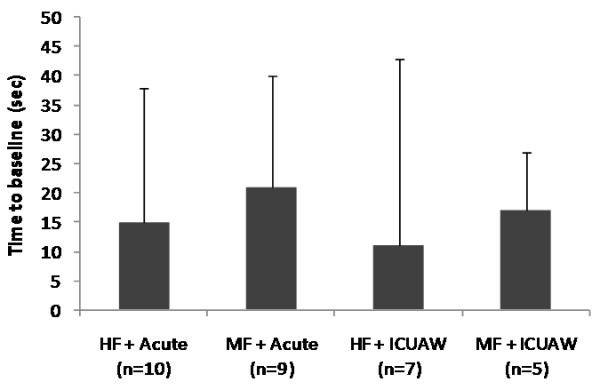
**Change scores in thenar muscle vascular reserve (time to baseline tissue oxygen saturation (StO**_**2**_**) value) as measured by near-infrared spectroscopy (NIRS)_during a vascular occlusion test for each neuromuscular electrical stimulation (NMES) protocol and patient group combination.** Scores are calculated as before minus after values.

The parameters of the systemic microcirculation before and after the NMES session for the acute and the ICUAW groups are shown in Table 4. In both patient groups, StO_2_ upslope and time to baseline were modified by the application of NMES in a statistically significant degree, while this was not the case for the StO_2_ downslope. The combined effects of NMES protocol and patient group in the measured microcirculatory parameters are shown In Figures 1, 2, 3 and 4.

**Table 4:** Changes in microcirculatory parameters monitored by near-infrared spectroscopy and use of a vascular occlusion test in the two patient groups

	**Acute group (n = 19)**			**ICUAW group (n = 12)**		
**Microcirculatory parameter**	**Before**	**After**	***P***	**Before**	**After**	***P***
Tissue oxygenation (StO_2_)	78 ± 11	77 ± 11	0.2	76 ± 8	75 ± 8	0.4
StO_2_ downslope (%/minute)	8.6 ± 3.4	9.1 ± 3.1	0.4	9.4 ± 2.6	10.3 ± 3.6	0.1
StO_2_ upslope (%/second)	2.4 ± 1.4	2.8 ± 1.5	0.03	2.9 ± 1.3	3.6 ± 1.8	0.04
Time to baseline (seconds)	160 ± 48	144 ± 47	0.003	162 ± 47	147 ± 34	0.04

### Current intensity and muscle contractions

The current intensity applied at the start of the NMES session was 57 ± 22 mA in the HF group and 63 ± 24 mA in the MF group (*P* = 0.4). By the end of the session the intensities had been raised to 87 ± 37 mA and 99 ± 36 mA, respectively (*P* = 0.3). The increase in current intensity that was achieved during the NMES session was 32 ± 18 mA with the HF protocol and 38 ± 17 mA with the MF protocol (*P* = 0.35 for the comparison of the two protocols). The muscle contractions that were elicited from the quadriceps muscle had a median rating of 2 and IQR of 2 with the HF protocol, and also a median rating of 2 and IQR of 2 with the MF protocol (*P* = 0.6 for the comparison of the two protocols).

When the two groups of patients were compared, patients in the acute group and those in the ICUAW group reached similar current intensities, with increases in intensity of 32 ± 18 mA and 40 ± 16 mA, respectively (*P* = 0.2 for the comparison). On the other hand, and in line with their diagnosis, patients in the ICUAW group developed less intense muscle contractions in the lower limbs than patients in the acute group for comparable current intensities: median 1.5 with IQR 2 versus median 3 with IQR 2 (*P* = 0.002).

The changes in the NIRS-derived parameters of StO_2_ upslope and time to baseline were used as outcome variables in regression models which had as a single predictor the following variables: NMES protocol, patient group (acute or ICUAW), final current intensity, increase in current intensity, strength of muscle contractions. In both cases, the strength of the muscle contractions was the variable that best predicted the changes of the parameter during the NMES session (Table 5).

**Table 5:** The Akaike Information Criterion for regression models with change in microcirculatory parameters during neuromuscular electrical stimulation (NMES) as the outcome variable, and those listed in each line of the table as the predictor

**Variable**	**StO**_**2 **_**upslope**	**Time to baseline**
NMES protocol	99.38	266.18
Patient group	94.78	266.43
Final current intensity	97.94	266.44
Increase in current intensity	96.38	265.75
Strength of muscle contractions	74.92	214.28

StO_2_, tissue oxygen saturation.

### NIRS measurements of the vastus lateralis

Of the 31 patients included in the study, only in seventeen were NIRS readings from the vastus lateralis technically feasible. In the rest, no NIRS signal could be obtained, despite the fact that during the design of the study we had anticipated the known factors that interfere with the NIRS readings. Patients with and without NIRS readings did not differ with respect to severity of illness (APACHE II score 19 ± 5 versus 17 ± 3, *P* = 0.5 and SOFA score 7 ± 2 versus 6 ± 3, *P* = 0.3) and presence of ICUAW (7/17 versus 5/14, *P* = 0.5). Patients with NIRS readings, however, had somewhat stronger muscle contractions with median 2 and IQR 2 versus median 1 and IQR 2 (*P* = 0.08). They also differed in their response in the thenar NIRS measurements: the StO_2_ upslope increased by 0.7 ± 0.9 versus 0.4 ± 0.8%/second, while time to baseline decreased by 18 ± 22 versus 14 ± 22 seconds.

In the patients with NIRS measurements (10 in the HF protocol and 7 in the MF protocol), the effects of NMES on the microcirculation of the exercising muscle did not differ between the two protocols. Baseline StO_2_ values in the vastus lateralis were 48 ± 19 units versus 51 ± 17 units, respectively (*P* = 0.7). ΔStO_2slope_ was 61 ± 6%/minute in the HF protocol and 69 ± 23%/minute in the MF protocol (*P* = 0.5). ΔStO_2min_ was 5 ± 4 units with the HF protocol and 7 ± 4 units with the MF protocol (*P* = 0.3). ΔStO_2max_ was 6 ± 7 units with the HF protocol and 5 ± 4 units with the MF protocol (*P* = 0.6).

All three measured microcirculatory parameters in the exercising muscle differed between the acute and the ICUAW group of patients. ΔStO_2slope_ was 85 ± 27%/minute in the acute group and 42 ± 13%/minute in the ICUAW group (*P* = 0.04). ΔStO_2min_ was 8 ± 4 units in the acute group and 3 ± 2 units in the ICUAW group (*P* = 0.005). ΔStO_2max_ was 8 ± 6 units in the acute group and 3 ± 2 units in the ICUAW group (*P* = 0.04).

The strength of the muscle contractions correlated well with the microcirculatory parameters (ΔStO_2slope_: r_s_ = 0.7 with *P* = 0.05; ΔStO_2min_: r_s_ = 0.8 with *P* < 0.001; ΔStO_2max_: r_s_ = 0.6 with *P* = 0.01).

## Discussion

In this study comparing the acute effects of two types of NMES, the main finding was that both medium frequency and high frequency pulsed current induced changes of similar magnitude on the systemic and local skeletal muscle microcirculation of critically ill patients. Moreover, the safety profile was equally good for the two types.

The application of NMES in critically ill patients is relatively new. There is evidence that NMES can prevent loss of muscle mass and strength in these patients [11-13,15]. This suggests that it could be a viable option for the prevention or the amelioration of ICUAW [14,35]. There is one study that showed systemic effects of a single NMES session on skeletal muscle microcirculation [16]. Microcirculatory alterations are a hallmark of sepsis, so these effects could mediate the beneficial effects observed in the skeletal muscle of septic patients.

One unresolved aspect of NMES use in the critically ill is the optimal frequency of the applied current. Existing studies have used frequencies at the medium (45 to 50 Hz) and the high range (70 to 100 Hz). The only study that examined acute systemic effects used a 45 Hz current [16]. There is no study that examines the systemic and local effects of both medium and high frequency currents. To the extent that their effects are mediated by effects on the microcirculation level, such a comparison is of considerable interest.

This study confirmed that NMES improved endothelial reactivity in a peripheral skeletal muscle not directly involved in the exercise. This effect has been ascribed to the systemic action of muscle-derived cytokines, mainly interleukin-6 (IL-6), whose levels are known to increase during exercise [36-38]. Both current frequencies displayed this ability to a similar degree.

The two protocols also demonstrated the capacity to improve the vascular reserve of the microcirculation. This is related to the magnitude of capillary recruitment after an ischemic challenge. Sympathetic activation and its effects on vascular tone might be at the origin of this phenomenon [39].

Local O_2_ consumption rate has been shown to increase during NMES in a previous study [16]. This is in line with other studies which have showed that NMES increases O_2_ uptake in healthy volunteers [40-42] and in COPD patients [43]. In fact, in a direct comparison of two types of NMES with frequencies of 15 Hz and 75 Hz both types of NMES were able to increase ventilation and O_2_ uptake in COPD patients [44]. In the present study we observed an increase of the local muscle O_2_ consumption rate of similar degree in both NMES protocols, and also in both patient groups. However, our observed effect was less than that reported by Gerovasili ***et al***. [16]. This might account for the lack of statistical significance in our findings, which come from a similar sample size.

A related finding of clinical relevance was that the degree of the changes in the microcirculation seems to be related to the quality of the muscular contractions. This would imply that any beneficial effects that NMES derive from the exercising muscle. It also advocates the timely application of NMES during critical illness.

As far as the local microcirculation of the exercising muscle is concerned, the results of this study were entirely analogous to the systemic case. NMES using pulsed currents of medium and high frequency have very similar effects.

ΔStO_2slope_ during a contraction reflects the oxidative energetic cost of muscle activation [32,45]. This interpretation rests on the assumption that an isometric contraction above 25 to 35% of maximal voluntary contraction blocks or severely limits arterial inflow and O_2_ delivery because of the increased intramuscular pressure [46]. The two protocols under study achieved muscle contractions of comparable strength, which is a plausible explanation for this finding. Although ours is the first study directly comparing two types of NMES, there are two previous studies by the same research group that have separately compared voluntary contractions with two NMES protocols using different frequencies for each comparison. They examined the biceps brachii muscle and they used a NIRS device identical to the one used in the present study to evaluate oxygenation. They concluded that local O_2_ demand during a NMES-induced isometric contraction of 30 seconds was similar in using a 30 Hz and a 75 Hz current [47,48].

ΔStO_2min_ values were also similar in the two protocols. This parameter reflects the dynamic equilibrium between O_2_ demand and O_2_ delivery to the volume of muscle illuminated by the NIRS probe. This equilibrium is affected by several factors such as the mechanical pressure on vessels during contraction, blood flow and consequently blood volume, venous return, relative degree of vasoconstriction and vasodilation in the microcirculation [49,50]. Our findings indicate that O_2_ consumption increased relatively to O_2_ delivery to a similar extent with the two types of current.

These parameters correlated well with the strength of muscle contractions. This can also be explained by the physiology of contraction and the transmission of pressures to the interior of the vasculature. It is also in agreement with a previous study which showed that O_2_ consumption estimated by NIRS in the biceps brachii during isometric contractions increases proportionally to the force developed from 20 to 80% of maximal voluntary contraction [32].

NMES showed an excellent safety profile in our study. Biochemical monitoring for muscle injury showed no adverse effects in an extended follow-up. This is in agreement with recent results that confirm the safety of NMES application [51,52].

The main limitation of this study was the small number of patients with NIRS measurements of the vastus lateralis muscle. These patients were clinically similar to those from whom a signal could be obtained; however, a difference was found in contracting ability. It remains to be clarified if the inability to detect a NIRS signal was due to some muscle characteristic that also interfered with contractions or with the passage of current through tissue. It is possible that this difference in contractile ability reflects local factors (for example, tissue edema) not clinically identifiable, but correlated with more diseased muscle and this cannot be captured by the conventional scales that measure disease severity, such as the SOFA score. Another limitation is that by design we examined only short-term effects of NMES on the microcirculation. These effects might well be short-lived, so that repeated applications of NMES might be required. Frequency of application and overall duration of NMES treatment were outside the scope of our protocol; further studies should address these issues.

## Conclusion

We could not find differences in the effects that a session of NMES with medium and high frequencies has on skeletal muscle microcirculatory function, both at the systemic and local level. Further studies are needed to compare the effects of the two types on clinical outcomes related to muscle function and the development of ICUAW.

## Abbreviations

AIC: Akaike information criterion; APACHE II: Acute physiology and chronic health evaluation II; CK: creatine kinase; COPD: chronic obstructive pulmonary disease; HF: high frequency; ICUAW: intensive care unit-acquired weakness; IL-6: interleukin-6; IQR: interquartile range; MF: medium frequency; NIRS: near-infrared spectroscopy; NMES: neuromuscular electrical stimulation; SIRS: systemic inflammatory response syndrome; SOFA: Sequential organ failure assessment; StO2: tissue oxygen saturation; VOT: vascular occlusion technique.

## Competing interests

The authors declare that they do not have competing interests.

## Authors’ contributions

EA contributed to the conception and design of the study, data acquisition, analysis and interpretation of the results, and drafted the manuscript; LK contributed to the design of the study, application of NMES, and critically revised the manuscript; SD contributed to the conception and design of the study and critically revised the manuscript; GM contributed to data acquisition and NMES application; CS contributed to the design of the study and data acquisition; IP contributed to the acquisition of data, and made the clinical assessments for ICUAW; AK contributed to the design of the study and to the interpretation of data; CR contributed to the interpretation of data and critically revised the manuscript; GP contributed to the conception of the study and critically revised the manuscript; SN contributed to the conception and design of the study, data interpretation, critically revised the manuscript and gave final approval of the version to be published.
